# O.6.3-10 Investigating the impact of a school garden intervention on children’s school motivation, climate literacy, food literacy and physical activity: the FoodACT protocol

**DOI:** 10.1093/eurpub/ckad133.290

**Published:** 2023-09-11

**Authors:** Anna Stage, Mads Bølling, Glen Nielsen, Peter Bentsen, Mette Aadahl, Camilla Roed Otte, Peter Elsborg

**Affiliations:** Center for Clinical Research and Prevention, Copenhagen University Hospital – Bispebjerg and Frederiksberg; Department of Nutrition, Exercise and Sports, University of Copenhagen; Center for Clinical Research and Prevention, Copenhagen University Hospital – Bispebjerg and Frederiksberg; VIA University College, Research Centre for Pedagogy and Bildung; Center for Clinical Research and Prevention, Copenhagen University Hospital – Bispebjerg and Frederiksberg; Center for Clinical Research and Prevention, Copenhagen University Hospital – Bispebjerg and Frederiksberg; Department of Geoscience and Natural Resource Management, University of Copenhagen, Rolighedsvej 23, 1958 Frederiksberg, Denmark; Center for Clinical Research and Prevention, Copenhagen University Hospital – Bispebjerg and Frederiksberg; Haver til Maver, Copenhagen; ansh@nexs.ku.dk; Center for Clinical Research and Prevention, Copenhagen University Hospital – Bispebjerg and Frederiksberg

## Abstract

**Background:**

Globally, too many children are not sufficient physically active and have unhealthy food habits with the highest prevalence seen in families with low socio-economic-status (SES). Previous research has stated that schools are considered a key setting for promoting children physical activity (PA), food literacy (FL) and to improve their social health. School gardens is exactly such an intervention where teaching is relocated to an outdoor setting. Relocation of teaching, which is a central ingredient of school gardens, has shown to be positively related to increased PA in both boys and girls during the school day. School gardens aim towards practical experience and education in food and climate. PA is not I focus, but the school garden learning environment provides pupils opportunities to be active. Therefore, the FoodACT study aims to investigate how a school gardening intervention impact pupils school motivation, CL, FL and PA with a special attention on children with low SES.

**Methods:**

The FoodACT study is designed in three sub-studies. An efficacy study, quantifying the effects of school gardens using a pre-post quasi-experimental design measuring the pupils’ FL, CL and SM. A within-subject-design association study, quantifying the influence of the school garden activities on pupils PA at two sessional periods during the intervention. An association study, investigating the contextual characteristics of the school garden activities using the systematic observations instrument, The Physical Activity Research and Assessment tool for Garden Observation (PARAGON), which can capture pupils’ movements and motions categorised in ‘overall PA-level’, ‘garden-related tasks’, ‘garden related motions’, ‘social associations’, and ‘interaction’. The observations is supplemented by pupil focus-group interviews about their experience of the school garden intervention.

**Results:**

Study design, recruitment experience and pilot-study data on PA and garden activity observations will be presented.

**Funding:**

The FoodACT study is funded by the Novo Nordisk Foundation (Grant no: 077522).

